# Acid Solution Processed VO_2_-Based Composite Films with Enhanced Thermochromic Properties for Smart Windows

**DOI:** 10.3390/ma14174927

**Published:** 2021-08-30

**Authors:** Zhe Wang, Bin Li, Shouqin Tian, Baoshun Liu, Xiujian Zhao, Xuedong Zhou, Gen Tang, Aimin Pang

**Affiliations:** 1 State Key Laboratory of Silicate Materials for Architectures, Wuhan University of Technology (WUT), No. 122, Luoshi Road, Wuhan 430070, China; 290952@whut.edu.cn (Z.W.); libin625@whut.edu.cn (B.L.); liubaoshun@126.com (B.L.); opluse@whut.edu.cn (X.Z.); xuedong_zhou@whut.edu.cn (X.Z.); 2 Science and Technology on Aerospace Chemical Power Laboratory, Hubei Institute of Aerospace Chemotechnology, Xiangyang 441003, China; tanggen518@126.com

**Keywords:** VO_2_, thermochromic properties, acid solution process, porous structure

## Abstract

As a typical thermochromic material, VO_2_ coatings can be applied to smart windows by modulating the transmission of near infrared (NIR) light via phase transition. However, the inherent undesirable luminous transmittance (*T*_lum_) and solar modulation efficiency (Δ*T*_sol_) of pure VO_2_ impede its practical application. In order to solve this problem, the porous VO_2_ based composite film was prepared by magnetron sputtering and subsequent acid solution process with Zn_2_V_2_O_7_ particles used as a sacrificial template to create pores, which showed excellent *T*_lum_ (72.1%) and enhanced Δ*T*_sol_ (10.7%) compared with pure VO_2_ film. It was demonstrated that the porous structure of the film caused by acid solution process could improve the *T*_lum_ obviously and the isolated VO_2_ nanoparticles presented strong localized surface plasmon resonance (LSPR) effects to enhance the Δ*T*_sol_. Therefore, this method will provide a facile way to prepare VO_2_ based films with excellent thermochromic performance and thus promote the application of the VO_2_ based films in smart windows.

## 1. Introduction

VO_2_ is an important thermochromic material with high potential for low-cost switching and energy saving devices which has been widely studied due to its reversible semiconductor to metal transition accompanied by a dramatic change in optical and electrical properties at near room temperature (~68 °C) [1]. With this characteristic, VO_2_ was usually investigated for thermochromic smart windows to reduce building energy consumption in response to the dynamic environmental temperature since significant decrease in the NIR transmittance and negligible changes in the visible and UV transmittance upon phase transition were exhibited. Therefore, it is of great significance to prepare VO_2_ based films with excellent thermochromic properties for smart windows. However, VO_2_ based thermochromic window is faced with great challenges of reducing the phase transition temperature (*T*_c_), improving durability, enhancing luminous transmittance (*T*_lum_) and solar modulation efficiency (Δ*T*_sol_) simultaneously. It is generally believed that the *T*_lum_ up to 60% and the Δ*T*_sol_ higher than 10% to be satisfied with the actual demand of smart windows applications [2,3,4,5,6].

In order to tackle the above issues, various methods have been employed such as establishing nano-composite film, multilayers and porous structures [6,7,8,9,10,11,12,13,14,15,16,17], doping [2,3,13,14,15] and optimization of preparation technology [10,11,18,19,20,21,22,23,24,25,26]. Kang et al. [13] and Jiang et al. [15] introduced Zn into VO_2_ lattice, which can improve the *T*_lum_ and Δ*T*_sol_ of the film, and at the same time faded the yellow color of the film, however the unsatisfied optical properties were far from application requirement. Chen et al. [7] dispersed VO_2_ nanoparticles into thermochromic nickel-bromine-ionic liquid (Ni-Br-IL) for enhancing optical performance. The composite film demonstrated desirable optical properties: *T*_l,lum_ = 65.9% and *T*_h,lum_ = 55.3% with extraordinary Δ*T*_sol_ of 27.0%. Wu et al. [16] introduced mesopores into the VO_2_ nanoparticles using cotton as the template by hydrothermal methods. The *T*_lum_ of mesoporous VO_2_ nanoparticles based composite film was up to 56.0%, a larger pore size could lead to a higher luminous transmittance. It was worth noting that *T*_lum_ and Δ*T*_sol_ for application of smart windows have to exceed 60% and 10% simultaneously. However, the most of the composite films prepared by the above methods have poor repeatability, durability as well as adhesive force to glass substrate. Magnetron sputtering method exhibited advantages of high repeatability; suitability for mass production; preparing coatings with uniform density, good durability and excellent Δ*T*_sol_. However, the much dense and smooth surface strongly increased the reflectance of the film, thus reducing the *T*_lum_ significantly. Taking appropriate approaches to increase the *T*_lum_ of film prepared by magnetron sputtering method is of great significance for taking full advantage of the magnetron sputtering method. According to this, Long et al. [26] used an acid solution to etch the pure VO_2_ film prepared by the magnetron sputtering, forming a karst landforms-like structure to improve the modulation efficiency and visible light transmittance of the VO_2_ film simultaneously. VO_2_ is quite easy to react with acid so that the porous structure obtained by the etching process is uncontrollable. In addition, after the acid solution process, the phase transition temperature of the film will be probably increased due to the reduce of interfacial strain [23,24,25], which is usually ignored and lack of detailed discussion. Therefore, it is of great importance to develop a facile method to prepare controllable porous structure of VO_2_ based film.

Inspired by this analysis, differing from directly etching pure VO_2_ film, we introduce Zn_2_V_2_O_7_ as the etching template into the VO_2_ film since the Zn_2_V_2_O_7_ possess higher activity than VO_2_ in the reaction with acid, which greatly improves the targeting of acid solution process, making the porous structure preparation in the film more controllable and avoiding more VO_2_ is corroded to guarantee the desirable Δ*T*_sol_. In this work, Zn_2_V_2_O_7_-VO_2_ composite films were prepared by magnetron sputtering followed by post annealing method using V and ZnO targets. The acid solution processed on the VO_2_ composite film aimed to generate porous structure by disposing of Zn_2_V_2_O_7_. The content of Zn_2_V_2_O_7_ in the composite film was adjusted to vary the porosity by changing the sputtering power of ZnO target. Consequently, porous VO_2_ film obtained by acid solution treatment showed the best optical performance (Δ*T*_sol_ = 10.7%, *T*_lum_ = 72.1%) when the sputtering power of ZnO target was set as 150 W. It is believed that the performance meets the requirement for application in smart windows.

## 2. Materials and Methods

**Figure 1:**
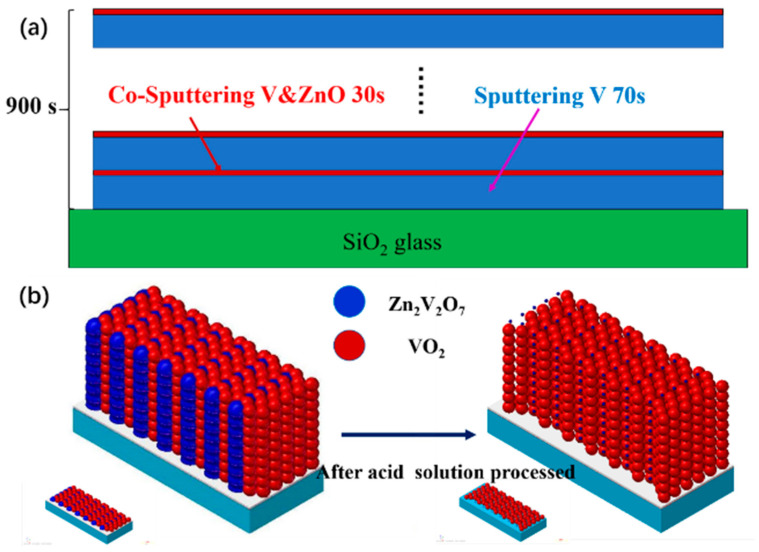
(**a**) the diagram for the ZnO-V composite film preparation process and (**b**) film structure before and after acid solution process.

All reagents were purchased from Sinopharm Chemical Reagent Co., Ltd. and used without further purification. The cleaned quartz glass substrate was fixed with heat-resistant tape in the magnetron sputtering vacuum chamber. V (99.95%) and ZnO (99.99%) targets were used for codeposition. Before sputtering, the vacuum chamber was evacuated to 3.0 × 10^−3^ Pa. Then, Ar (99.99%) was introduced into the chamber and the gas flow rate was fixed at 200 sccm. First, ZnO-V composite films were deposited through direct current magnetron sputtering of V targets at power of 90 W and radio frequency magnetron sputtering of ZnO targets simultaneously at various power. The total continuous sputtering duration of V target was 15 min while the zinc oxide target was intermittently sputtered 9 times with 30 s for each time. The detailed fabrication process of ZnO-V composite films was illustrated in Figure 1a. Finally, Zn_2_V_2_O_7_-VO_2_ composite films was achieved through post annealing the ZnO-V composite films in tube furnace. Detailly, the films were put into a tube furnace with air pressure of 1000 Pa. Ramping up the temperature to 450 °C and holding for 1 h at a rate of 5 °C/min, and then cooling down naturally. For comparison, ZnV_2_O_7_-VO_2_ composite films with different sputtering power of ZnO were denoted as sample a1 (60 W), b1 (90 W), S1 (120 W) and c1 (150 W), respectively, and pure VO_2_ film without sputtering ZnO was also prepared.

The Zn_2_V_2_O_7_-VO_2_ composite films were put into a PTFE etching flower basket and treated in hydrochloric acid with a molar concentration of 5.6 mol/L for 5 s. After corrosion, films were quickly removed out of the acid solution and ultrasonic cleaned in deionized water and absolute ethanol for 1 min. The as-obtained porous VO_2_ films were blow dried with N_2_ and denoted as sample a2 (60 W), b2 (90 W), S2 (120 W) and c2 (150 W) corresponding to the Zn_2_V_2_O_7_-VO_2_ composite films respectively. The schematic diagram of films structure evolution before and after acid solution treatment were shown in Figure 1b.

Glancing-angle X-ray diffraction (GAXRD) measurements were used to characterize the crystal structure of the films on an Empyrean diffractometer (Cu Kα, *λ* = 0.154178 nm produced under a 4kW output power, Malvern Panalytical B.V., Almelo, Netherlands). Field emission scanning electron microscope (SEM, JSM-5610LV, JEOL, Tokyo, Japan) with X-Max 50 X-ray energy spectrometer and atomic force microscopy (AFM, Nanoscope IV/Nanoscope IV, VEECO, New York, NY, USA) were adopted to observe the morphology of the films. X-ray photoelectron spectroscopy (XPS, ESCALAB 250Xi/ESCALAB 250Xi, Thermo Fisher, Waltham, MA, USA) was employed to determine the element composition of the films. An ultraviolet-visible-near-infrared spectrophotometer (UV-3600, Shimadzu, Kyoto, Japan) was used to test the solar transmittance of the films in the range of 300–2500 nm at 20 and 90 °C, respectively. The integrate *T*_lum_ and *T*_sol_ can be calculated by the following Equations (1) and (2).

(1) $$T_{\mathrm{lum}}=\int_{380}^{780}\nu_{m}\left(\lambda \right)T\left(\lambda \right)d\lambda /\int_{380}^{780}\nu_{m}\left(\lambda \right)d\lambda$$

(2) $$T_{\mathrm{sol}}=\int_{300}^{2500}\varphi_{\mathrm{sol}}\left(\lambda \right)T\left(\lambda \right)d\lambda /\int_{300}^{2500}\varphi_{\mathrm{sol}}\left(\lambda \right)d\lambda$$

The Δ*T*_sol_ can be calculated by Equation (3).

(3) $$T_{\mathrm{sol}}=T_{\mathrm{sol}}\left(20\;{}^{\circ}C\right)-T_{\mathrm{sol}}\left(90\;{}^{\circ}C\right)$$

where *T*(λ) is the transmittance, λ is the wavelength of the incident light, *ν*_m_(λ) denotes the spectral sensitivity of the light to the human eye and *φ*_sol_(λ) represents the irradiance spectrum of the sunlight at an atmospheric mass of 1.5 (corresponding to the sun from the horizon 37°) [27,28,29,30,31,32,33,34,35,36,37].

## 3. Results and Discussion

### 3.1. Structures of the Films before and after Acid Solution Treatment

**Figure 2:**
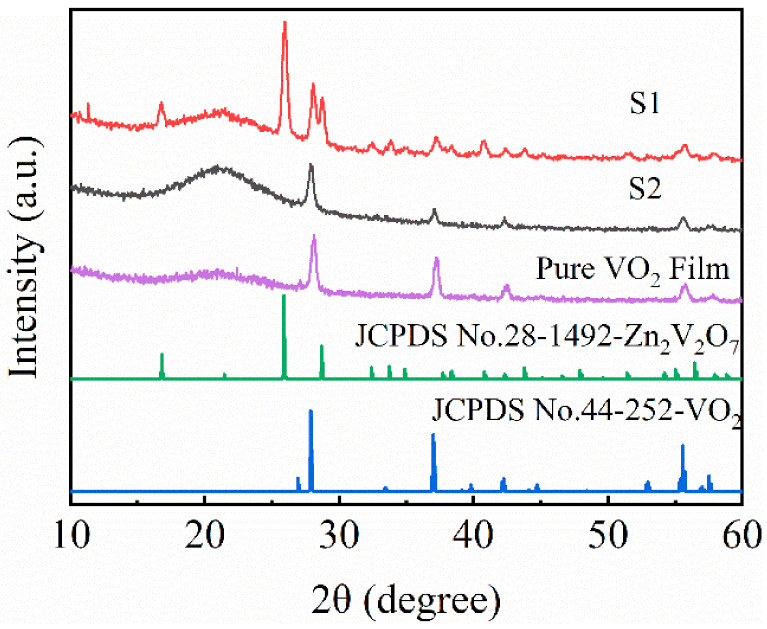
XRD patterns of sample S1, S2 and pure VO_2_ film.

The XRD patterns of the samples before (sample S1) and after (sample S2) the acid solution process as well as the pure VO_2_ film were shown in Figure 2 (and Figure S1). It can be seen that sample S1 was consist of VO_2_(M) and Zn_2_V_2_O_7_ corresponding to the standard JCPDS card No. 44–252 and 28–1492, respectively. This indicates that ZnO-V composite films were fully transferred to Zn_2_V_2_O_7_-VO_2_ composite films with a reaction of ZnO and VO_2_. Besides, the relative intensity of the VO_2_ diffraction peaks in the Zn_2_V_2_O_7_-VO_2_ composite film were lower than those in the pure VO_2_ film, implying that some of the VO_2_ was consumed upon the reaction. After acid solution process, only diffraction peaks of VO_2_ were observed in sample S2 and the relative intensity decreased, which indicated that Zn_2_V_2_O_7_ as the template were totally corroded. It was worth noting that the relative intensity of VO_2_ diffraction peaks remained unchanged. Consequently, porous structures could be formed through sacrifice of some Zn_2_V_2_O_7_ particles as expected. In addition, there is a shift in the peak position of the (011) reflection of M-VO_2_ among sample S1, S2 and pure VO_2_ film, indicating that there are some strains in the samples.

**Figure 3:**
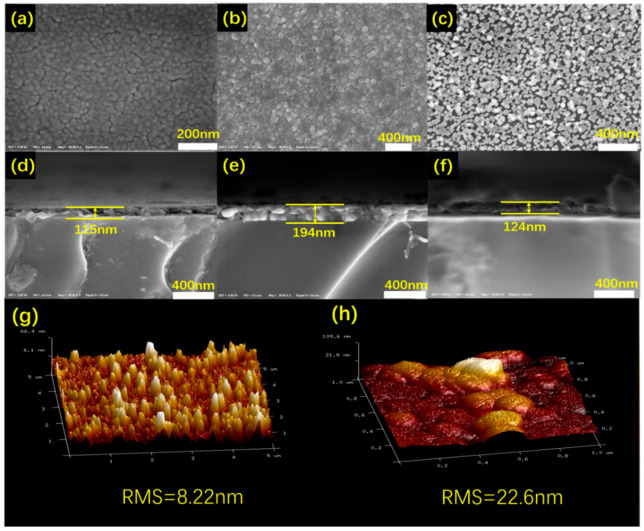
Top view SEM images of surface morphology of (**a**) pure VO_2_ film, (**b**) sample S1 and (**c**) sample S2, cross-section SEM images of (**d**) pure VO_2_ film, (**e**) sample S1 and (**f**) sample S2 and AFM images of (**g**) sample S1 and (**h**) sample S2.

In order to further investigate the effect of acid solution treatment on the morphologies evolution of the samples, SEM and AFM test were performed on sample S1, S2 and pure VO_2_ film and the results were depicted in Figure 3. It can be seen that the pure VO_2_ film and sample S1 were quite dense that was comparable to the films prepare by magnetron sputtering method which was unfavorable for the optical properties. Meanwhile the color of top view of the films surface evolved from dark to bright for pure VO_2_ film, sample S1 and sample S2 gradually, which means the roughness of films increased as the SEM images are morphological liner images [33], and the deduction were further confirmed by the AFM images shown in Figure 3g,h in which the root mean-square roughness (RMS) was obviously increased from 8.22 nm of sample S1 to 22.6 nm of sample S2. With introduction of Zn_2_V_2_O_7_, the grains in sample S2 became less uniform. After acid solution treatment, the film was the brightest. It can be clearly seen that the porous structures between the isolated particles in the films increased significantly after acid solution treatment (Figure 3c) since the Zn_2_V_2_O_7_ were removed by the acid solution. It was believed that such typical structures would benefit for improvement of *T*_lum_ and Δ*T*_sol_ simultaneously since the LSPR effect would generate. Additionally, the film thickness increased significantly from 115 nm to 194 nm for pure VO_2_ film and Zn_2_V_2_O_7_-VO_2_ composite film (S1) due to the generation of Zn_2_V_2_O_7_ (Figure 3d–f). After acid solution treatment on sample S1, film thickness for sample S2 reduced to 124 nm that was almost identical to that of the pure VO_2_ film, indicating the Zn_2_V_2_O_7_ was performed as the sacrificial template to prepare the porous structures with VO_2_ being isolated (Figure 3c).

**Figure 4:**
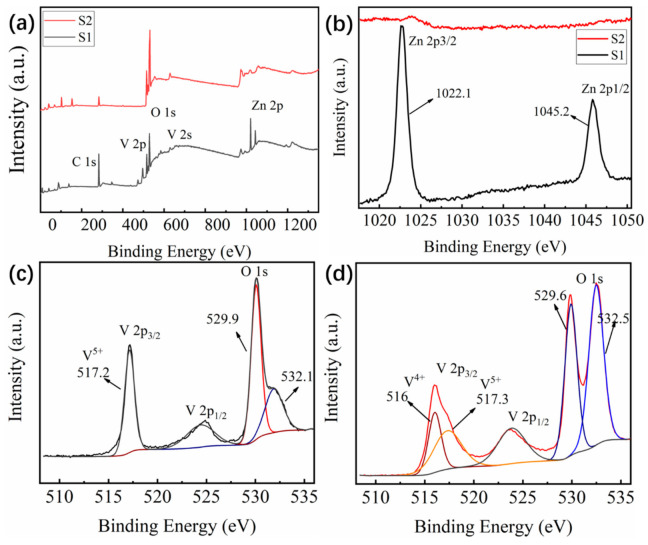
(**a**) XPS survey spectrum of samples S1 and S2, high-resolution XPS spectra for (**b**) Zn 2p, V 2p and O 1s core levels in (**c**) sample S1 and (**d**) sample S2.

In order to analyze the elemental composition in the films, XPS characterization was performed and the results are shown in Figure 4. All the core level binding energies have been calibrated by the standard C 1s binding energy of 284.8 eV. The Zn 2p peak was obviously observed in sample S1 but hardly seen in sample S2, indicating that there is a large amount of Zn in sample S1 while small amount of Zn in sample S2 (Figure 4a,b). Especially in Figure 4b, the Zn 2p core level was split into Zn 2p3/2 (1022.1 eV) and Zn 2p1/2 (1045.2 eV) peaks, indicating that Zn element in the samples existed as +2 in valence. It was demonstrated that the Zn_2_V_2_O_7_ was sacrificed completely after acid solution process, which agreed well with the above XRD and SEM results. Figure 4c,d showed the high-resolution analysis of V 2p_3/2_ and O 1s peaks, and the fitting curves based on Gaussian function. It can be seen that V element was mainly in a +5 valence state before acid solution process due to the formation of Zn_2_V_2_O_7_ on the surface of the Zn_2_V_2_O_7_-VO_2_ composite film (S1) according to the co-sputtering diagram in Figure 1a [38]. What is more, the XPS characterization could be only performed in the range of 2 nm in depth from the surface. O 1s core level (Figure 4c) was divided into two peaks that located at 529.9 and 532.1 eV, respectively. The former was assigned to the lattice oxygen in sample S1 while the latter belonged to the adsorbed oxygen species. After acid solution process was conducted in Zn_2_V_2_O_7_-VO_2_ composite film, both core level peaks for V 2p and O 1s changed obviously (Figure 4d). Comparing with sample S1, core level peak at 516 eV corresponding to V^4+^ appeared with considerable intensity. Meanwhile the position of two O 1s peaks were slightly shifted, indicating that the chemical environment of O^2−^ changed, as a result, the peak located at 529.6 eV was assigned to the lattice oxygen in sample S2 and another peak at 532.5 eV was indexed to adsorbed oxygen species since more oxygen species were adsorbed on the film surface due to porous structures in sample S2 [13,15,20]. In order to conduct a more in-depth study on the composition of the Zn_2_V_2_O_7_-VO_2_ composite films before and after acid solution process (sample S1 and S2), the quantitative analysis was carried out by the XPS and EDS (energy dispersive spectrometer) characterizations, and the results are shown in Table 1. It can be seen that after acid solution process, the content of Zn in the film was decreased significantly.

### 3.2. Thermochromic Properties of VO_2_ Based Films

**Figure 5:**
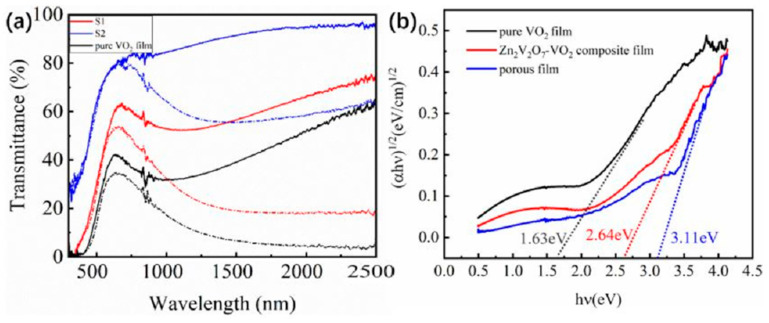
(**a**) the transmittance of sample S1, S2 and pure VO_2_ film at 20 (line) and 90 °C (short point line). (**b**) (αhν)1/2 and hν plots for VO_2_, Zn_2_V_2_O_7_-VO_2_ composite film, and porous film, indicating the optical band gap of each film.

Figure 5a showed the solar transmittance at 20 and 90 °C for sample S1, S2 and pure VO_2_ film. It can be seen that the transmittance of ZnV_2_O_7_-VO_2_ composite film (S1) and porous VO_2_ film (S2) was significantly increased compared with the pure VO_2_ film. For quantitative investigating the optical properties, the integrate luminous transmittance (*T*_lum_) and solar modulation efficiency (Δ*T*_sol_) were calculated. The Δ*T*_sol_ of the pure VO_2_ film obtained by magnetron sputtering is 11.6%, but *T*_lum_ was only 31.4%. By introducing Zn_2_V_2_O_7_ into VO_2_ film, both Δ*T*_sol_ and *T*_lum_ were improved to 14.0% and 45.0% for sample S1, respectively. Such improvement was attributed to the Zn_2_V_2_O_7_ particles dispersed in the VO_2_ film which did not absorb visible light due to their higher optical band gap compared with pure VO_2_ film in Figure 5b [30]. After acid solution process, the Δ*T*_sol_ of sample S2 was 10.7%, and the *T*_lum_ was as high as 72.1%, which is higher than previous reports [13,14,15,16,17,18,35,36,37,38,39,40]. The improvement in *T*_lum_ was probably due to the increased optical band gap induced by the reduced strain in the porous film compared with pure VO_2_ and the increase porosity in the films. It can be seen from Figure 3 that the porosity of the film is significantly increased after the acid solution process, and the size of the particle was slightly reduced in Table 2, which may increase the *T*_lum_ of the film. In addition, after acid solution process, some VO_2_ particles would be corroded, and the film become thinner, which will cause the Δ*T*_sol_ of the film to drop significantly. However, because the rest of the VO_2_ particle size became smaller, it will lead to strong LSPR effect [33,40], resulting in the enhancement of Δ*T*_sol_. Therefore, due to the influence of these two factors, the reduction of film modulation efficiency is not obvious.

**Figure 6:**
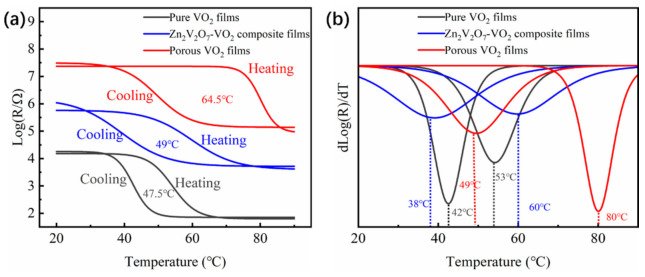
(**a**) the electrical hysteresis loop and (**b**) the corresponding differential for temperature of pure VO_2_ film, Zn_2_V_2_O_7_-VO_2_ composite films and porous film.

To determine the phase transition temperature (*T*_c_) of sample S1 and S2, the resistance of the film was recorded at gradient temperatures from 20 °C to 90 °C and the results are shown in Figure 6. Here, *T*_c_ is the average between the minimum in the differential resistance (*T*_heating_ + *T*_cooling_)/2. The *T*_c_ is 47.5 °C, 49 °C and 64.5 °C for the pure VO_2_ film, Zn_2_V_2_O_7_-VO_2_ composite film and porous VO_2_ film, respectively. These values of *T*_c_ were higher than the critical temperature of bulk VO_2_ (68 °C) because there are some strains in the obtained samples probably induced by the interface between films and substrates [35,37]. For pure VO_2_ film, the interface between VO_2_ particles can also leads to large strain, thus reducing the phase transition temperature. After the sacrifice of VO_2_ particles and production of Zn_2_V_2_O_7_ particles, the interface strain induced by particles was slightly reduced and the phase transition temperature was slightly increased to 49 °C. After the acid solution process, the interface strain between the Zn_2_V_2_O_7_ and VO_2_ particles was decreased obviously, hence the *T*_c_ was increased to 64.5 °C. Additionally, the hysteresis width got wider since the particle surface which was defects-enriched has been corroded, thus leading to the reduced concentration of defects. 

**Figure 7:**
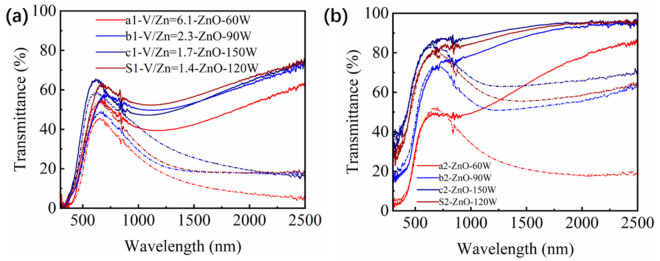
The transmittance of (**a**) Zn_2_V_2_O_7_-VO_2_ composite films: a1, b1, c1 and S and (**b**) porous VO_2_ films obtained by acid solution treatment of Zn_2_V_2_O_7_-VO_2_ composite films: a2, b2, c2 and S2 at 20 (line) and 90 °C short point line, respectively.

**Figure 8:**
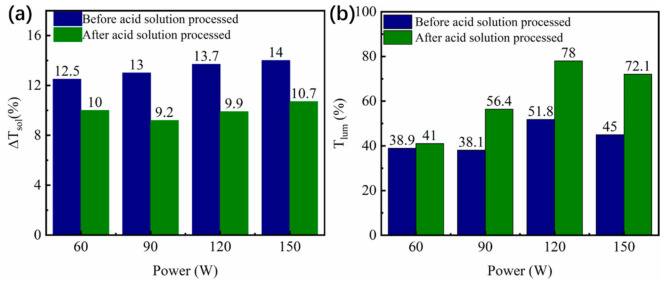
Comparison of (**a**) Δ*T*_sol_ and (**b**) *T*_lum_ between Zn_2_V_2_O_7_-VO_2_ composite films (before acid solution processing) and porous VO_2_ films (after acid solution processing) with different sputtering power of ZnO target.

**Figure 9:**
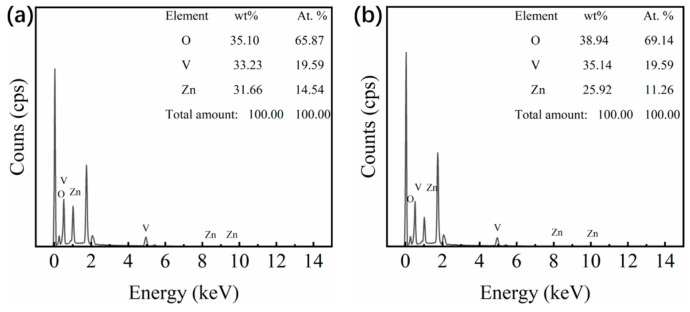
EDS (energy dispersive spectrometer) spectrum of (**a**) sample S1 and (**b**) sample c1 and the corresponding atomic and mass ratio for O, V and ZnO elements.

As expected, the Zn_2_V_2_O_7_ served as the sacrificial templates to generate the pores in the VO_2_ films. Therefore, the content of Zn_2_V_2_O_7_ was varied to evaluate the effect of porosity on the optical properties. As such, the sputtering power for ZnO target was set as 60 W, 90 W, 120 W and 150 W, respectively, to prepare Zn_2_V_2_O_7_-VO_2_ composite films with various Zn_2_V_2_O_7_ contents, and then porous VO_2_ films with different porosities were obtained by processing those composite films with acid solution in the same condition. Figure 7 shows the solar transmittance of different films. It can be seen that transmittance was obviously increased for both the Zn_2_V_2_O_7_-VO_2_ composite films and porous VO_2_ films as increasing the sputtering power of ZnO target. The quantitative optical properties were summarized in Table 2 and comparison between them was presented in Figure 8. It was worth noting that the sputtering power of ZnO, basically, represented the content of Zn_2_V_2_O_7_ since the Zn_2_V_2_O_7_ was generated by the reaction of ZnO and VO_2_, but sample S1 with sputtering power of 150 W was an exception. Comparing Zn content in both sample S1 and c1 (Figure 9), it could be found that the Zn content was decreased with increasing sputtering power from 120 W to 150 W since the Ar^+^ bombarded the target material too fast when power is high enough, thus some Zn^2+^ failed to deposit on the glass substrate. Therefore, the *T*_lum_ of the Zn_2_V_2_O_7_-VO_2_ composite films and porous VO_2_ films were considered to be linearly dependent on the content of Zn_2_V_2_O_7_ (Figure 8b). This is also consistent with the grain size trend in Table 2. After acid solution process, the replacement of Zn_2_V_2_O_7_ with pores would significantly improve the *T*_lum_ as the refractive index of Zn_2_V_2_O_7_ is much higher than that of air, and the *T*_lum_ was increased by the porosity in VO_2_ films. As the Zn content was increased, the grain size of the film increased slightly, but more Zn_2_V_2_O_7_ was produced, leading to an enhancement of *T*_lum_. The highest *T*_lum_ was 78% for sample c2, showing 50.6% improvement from sample c1, and for sample S1 and S2, the increment of 60.2% was obtained. The Δ*T*_sol_ was slightly decreased after acid solution process the Zn_2_V_2_O_7_-VO_2_ composite films because that the film thickness and content of VO_2_ were reduced after corroding in acid solution even though the LSPR effects appeared (Figure 6b). Nevertheless, the porous film (S2) exhibited the outstanding optical performance that meets well with the needs of practical application, especially for the magnetron sputtering prepared film, and also showed an obvious advantage in the luminous transmittance compared with other thermochromic films in Table 3. 

## 4. Conclusions

In this work, magnetron sputtering was used to prepare Zn_2_V_2_O_7_-VO_2_ composite films and the porous VO_2_ films were obtained by corroding the Zn_2_V_2_O_7_ that serve as the target sacrificial template in acid solution. Compared with processing the pure VO_2_ film, this method was highly targeted, and thus leading to the controllable porous structure in VO_2_ film. The porosity was linearly dependent on the content of Zn_2_V_2_O_7_ in Zn_2_V_2_O_7_-VO_2_ composite films after acid solution process. Hence, the *T*_lum_ was increased significantly with the increment of content of Zn_2_V_2_O_7_. When the ZnO sputtering power was 120 W and 150 W, the porous VO_2_ films obtained by acid solution process exhibited excellent optical performance with Δ*T*_sol_ of 9.9% and 10.7% and *T*_lum_ being up to 78% and 72.1%, respectively. Therefore, the combination of these two processes is of great significance for shedding light on the modification of VO_2_ films, and furthermore promoting the application of VO_2_ films on energy saving smart windows.

## Figures and Tables

**Table 1:** Elemental content of sample S1 and S2 based on the XPS and EDS results.

Element	XPS Results (at. %)		EDS Results (at. %)	
	S1	S2	S1	S2
V	30.5	35.4	19.6	7.09
Zn	11.7	0.5	14.54	0.03
O	57.8	64.1	-	-

**Table 2:** Optical properties and grain size of Zn_2_V_2_O_7_-VO_2_ composite films (before acid solution processing) and porous VO_2_ films (after acid solution processing) with different sputtering power of ZnO target.

Samples	Sputtering Power of ZnO Target (W)	Before Acid Solution Process V/Zn	Zn_2_V_2_O_7_-VO_2_ Composite Films			Porous VO_2_ Films		
			Grain Size (nm)	*T*_lum_ (%)	Δ*T*_sol_ (%)	Grain Size (nm)	*T*_lum_ (%)	Δ*T*_sol_ (%)
a	60	6.1	20.3	38.9	12.5	18.6	41.0	10.0
b	90	2.3	23.4	38.1	13.0	19.0	56.4	9.2
S	120	1.4	28.1	51.8	13.7	19.5	78.0	9.9
c	150	1.7	25.6	45.0	14.0	17.5	72.1	10.7

The grain size of pure VO_2_ film is 17.6 nm.

**Table 3:** Comparison of thermochromic performance of this work with previous works.

	Thermochromic Properties		Reference
System	*T*_lum_ (%)	Δ*T*_sol_ (%)	
Si doped VO_2_	-	9.2	Wu et al. [2]
Ni-Br-IL composite film	65.9	27.0	Chen et al. [7]
Zn-doped VO_2_	41.3	15.3	Kang et al. [13]
Mesoporous VO_2_ based film	56	12.9	Wu et al. [16]
two-dimensional nanostructure VO_2_ film	61.3	11.9.	Long et al. [26]
W-doped VO_2_ film	61.7	11.7	Zhang et al. [31]
Terbium-doped VO_2_ film	54.0	8.3	Wang et al. [34]
Porous VO_2_ film	72.1	10.7	This work

“-” Means data not mentioned.

## Data Availability

Not applicable.

## Supplementary Materials

The following are available online at https://www.mdpi.com/article/10.3390/ma14174927/s1, Figure S1: XRD patterns of Zn_2_V_2_O_7_-VO_2_ composite films (before acid solution processing) and porous VO_2_ films (after acid solution process) with different sputtering powers of ZnO target.
