# Supplementary material for: Acid Solution Processed VO2-Based Composite Films with Enhanced Thermochromic Properties for Smart Windows
Source: Materials (Basel). 2021 Aug 30;14(17):4927. doi: 10.3390/ma14174927 (PMC8434034; doi:10.3390/ma14174927)
Supplement: Supplementary file 1 [file materials-14-04927-s001.zip › materials-1318006-supplementary.pdf]

Supporting Information

Acid Solution Processed VO_2-Based Composite Films with Enhanced Thermochromic Properties for Smart Windows

Zhe Wang [1,2], Bin Li [1], Shouqin Tian [1,*], Baoshun Liu [1], Xiujian Zhao [1], Xuedong Zhou [1], Gen Tang [2] and Aimin Pang [2,*]

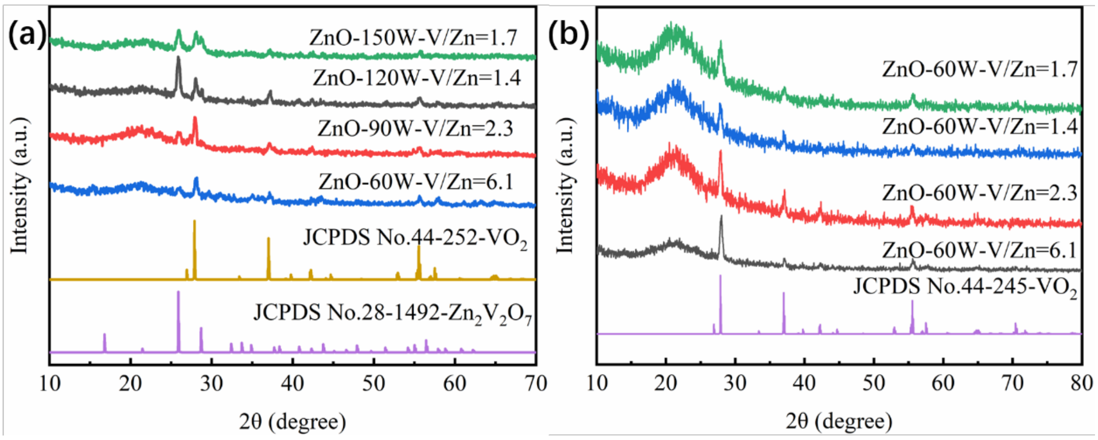

Figure S1. XRD patterns of $Zn_2V_2O_7$-VO_2 composite films ((**a**) before acid solution processing) and porous VO_2 films ((**b**) after acid solution process) with different sputtering powers of ZnO target.
